# Neutrophils Respond Selectively to Physical Cues: Roughness Modulates Its Granule Release, and NETosis

**DOI:** 10.3390/jfb15110342

**Published:** 2024-11-13

**Authors:** Gayathiri Elangovan, Daniel J. Fernandes, Andrew Cameron, Souptik Basu, Joao Martins De Mello Neto, Peishan Jiang, Peter Reher, Stephen Hamlet, Carlos Marcelo S. Figueredo

**Affiliations:** 1 School of Medicine and Dentistry, Griffith University, Brisbane, QLD 4101, Australia; gayathiri.elangovan@alumni.griffithuni.edu.au (G.E.); d.fernandes@griffith.edu.au (D.J.F.); a.cameron@griffith.edu.au (A.C.); s.basu@griffith.edu.au (S.B.); joao.martinsdemelloneto@jcu.edu.au (J.M.D.M.N.); anna.jiang@alumni.griffithuni.edu.au (P.J.); p.reher@uq.edu.au (P.R.); s.hamlet@griffith.edu.au (S.H.); 2 Department of Dental Medicine, Karolinska Institutet, 171 77 Solna, Sweden

**Keywords:** neutrophils, titanium oxide, zirconia, roughness

## Abstract

Our study examined how different titanium alloy Ti6Al4V (Ti64) and zirconia (ZrO_2_) surfaces, ranging from rough to very smooth, affect the expression of elastase (NE), matrix metalloproteinase (MMP)-8, MMP-9, and extracellular traps (NETs) by neutrophils. Discs of Ti64 and ZrO_2_, 10 mm in diameter and 1.5 mm thick, were created using diamond-impregnated polishing burs and paste to produce rough (Ra > 3 µm), smooth (Ra ≥ 1 to 1.5 µm), and very smooth (Ra < 0.1 µm) surfaces. Neutrophils from Wistar rats were cultured on these surfaces, and the culture supernatants were then examined for NE, MMP-8, and MMP-9 using ELISA. At the same time, NET formation was demonstrated immunohistochemically by staining neutrophils with CD16b and DNA with DAPI. Overall, the expressions of NE and MMP-8 were significantly higher from neutrophil culture on Ti64 and ZrO_2_ rough surfaces compared to the very smooth surface (R > S > VS) after 2 h and 4 h of culture. The expression of MMP-9 also increased with culture time; however, no significant surface effects on expression were observed. Similarly, rough Ti64 and ZrO_2_ surfaces (R & S) also showed significantly larger NET formation compared to the very smooth surface (VS) after 4 h and 8 h cultures. Our findings suggest that increasing surface roughness on Ti64 and ZrO_2_ triggers higher NE, MMP-8, and NET formation secretion.

## 1. Introduction

Titanium alloy Ti6Al4V (Ti64) or zirconia (ZrO_2_) implants have significantly improved patients’ rehabilitation over the past few decades [1,2]. However, implanting can lead to adverse immune reactions dominated by innate immune cells. This can result in excessive inflammation, impaired healing, fibrotic encapsulation, and tissue destruction [3]. These effects are triggered by various characteristics of implant surfaces, such as energy, chemistry, and topography [4,5,6]. It has been found that the roughness and chemistry of implant surfaces significantly influences cellular and physiological processes, including cell adhesion density, spreading and motility, proliferation and differentiation, and cytokine secretion [7,8].

The surface of titanium substrates forms a spontaneous and protective oxide layer instantaneously, which responds for the well-known corrosion resistance and biocompatibility of titanium and its alloys during applications as biomaterials. At atmospheric pressure, the titanium dioxide layer exists as amorphous or at any ratio among its three polymorph variants possible [9]. The chemical composition of the oxide layer from titanium alloy substrates partially reflects the composition of the bulk underneath, with mostly elemental distribution composed by oxygen and aluminium when the oxide layer experienced an increase in thickness [10].

Neutrophils are one of the first members of the innate system to arrive at the implantation site of biomaterials [11,12]. This group of cells is the first lineage of circulating immune cells to respond to tissue injuries or against infections [12,13]. They are generally associated with the acute inflammatory response, and depending on the extent of the injury on-site, the initial acute inflammation is turned into a chronic stage that can remain for weeks, depending on the arrival of mononuclear cells such as monocytes and lymphocytes [14]. Monocytes usually arrive at the implantation site and differentiate into macrophages, which have been exhaustively investigated in the literature [14,15,16]. However, macrophages are a ‘second line’ of defence of the body. Surprisingly, the influence of the neutrophils in the immune response and their ability to potentially mitigate early stages of inflammation and wound healing at implant sites remains to be fully delineated.

Neutrophils, when activated, undergo a rapid tenfold increase in circulating numbers. A defining characteristic of neutrophils is their remarkable ability to alter their shape swiftly. While in the bloodstream, neutrophils exhibit a near-spherical shape, which allows them to navigate narrow capillaries. Upon receiving the signal to exit the bloodstream and enter the tissue, they transition to a flattened shape on the endothelium surface. Upon reaching infection sites, neutrophils eliminate invading microbes through phagocytosis and the production of reactive oxygen species within the phagosome containing the microbe. Neutrophils can internalise pathogens or cell debris, recruit other immune cells, and release granular enzymes, reactive oxygen species, or nuclear extracellular traps (NETs) [11,16]. This study focused on measuring elastase, MMP-8, and MMP-9 to examine the release of primary, secondary, and tertiary granules. 

It is postulated that neutrophils follow phagocytosis or NET formation based on distinguishing the size of pathogens, which are small microbes or particles internalised. In contrast, the NETosis pathway neutralises large ones [11,15,17]. Thus, we hypothesised that neutrophils’ ability to sense the environment may also be responsive to differences across the topography of an implant surface. This would further regulate the immune response by modulating neutrophil enzyme secretion in the early stages of the healing period following implantation procedures. This experiment aims to validate if neutrophils are sensitive to differences in the degree of surface roughness of titanium oxide and zirconia substrates and if this stimulus can affect neutrophil degranulation and NETosis.

## 2. Materials and Methods

Ti64 and ZrO_2_ discs (98.5 mm in diameter and 14–20 mm in height) for the Colado CAD milling system (Ivoclar Vivadent, Schaan, Liechtenstein) were used as sources to design sample discs with cylindrical shape (10 mm in diameter and 1.5 mm in height). Ivoclar IPS e.max ZirCAD MT Multi Disc (Schaan, Liechtenstein) presents two different Yttrium oxide contents ranging from 4Y-TZP to 5Y-TZP.

Our samples were designed using an open-source computer design software version 3.5 (Meshmixer, Autocad) and milled using a 5-axis milling machine (Ivoclar Programill PM3, Schaan, Liechtenstein). Zirconium oxide discs were sintered at 1600 °C for 2 h in the Programat 7 Furnace (Ivoclar Vivadent Pty Ltd., Liechtenstein, Germany). Different grades of roughness were prepared by sequential polishing routes, including diamond-impregnated burs and polishing with diamond paste. Our recently published article provides a detailed procedure for preparing Ti64 and ZrO_2_ surfaces [18]. Three levels of roughness were created for each TiO_2_ and ZrO_2_ sample: rough (R) with Ra ≥ 3 µm, smooth (S) with Ra ≥ 1 to 1.5 µm, and very smooth (VS) with Ra ≤ 0.1 µm [18,19]. The morphology of the surfaces was analysed using a scanning electron microscope JEOL IT-300 (Jeol Ltd., Tokyo, Japan) at an accelerating voltage of 15 kV with a stage at perpendicular orientation. Samples were submitted to gold sputtering. A profile from each surface topography was registered using a 3D optical profilometer Zeta 300 3D (KLA Ltd., Milpitas, CA, USA). Different roughness descriptors were measured using an ×10 lens under a 100 µm × 100 µm scanning area. We measured Ra in five different regions in each disc (right, left, centre, top, and bottom), each value being an average of five other different linear profiles. A Z range of 50 µm mode was chosen, and each sample was scanned three times at five different spots: the centre, 3 mm right, 3 mm left, 3 mm below, and 3 mm above from the centre area. Mean roughness (Ra), root means square roughness (Rq), and mean maximum height of the profile (Rz) were calculated based on eight different measurements in a scanned area using the built-in software.

In total, 120 discs were generated, 60 from titanium and 60 from zirconium oxide. Each material had three groups (each n-10): rough (R), smooth (S), and very smooth (VS). These milled Ti64 and ZrO_2_ discs (10 mm diameter) were processed using different diamond-impregnated polishing burs (alphabond) and finally finished by polishing with diamond paste to generate a different grade of roughness which is clinically applicable. The discs were steam-cleaned with demineralised water and sterilised using an autoclave at 120 °C for 20 min.

Neutrophils were isolated from five 12-week-old male Wistar rats (Animal Resource Centre, WA, Australia) following ethics committee approval protocol by the Griffith University Ethics Committee (DOH/02/20/AEC) and also with ARRIVE guidelines 2.0. The institutional and national guidelines for the care and use of the animals in the study have been followed. The rats were euthanised using isoflurane, followed by cervical dislocation. Five ml of blood was collected through cardiac puncture, and neutrophils were isolated using histopaque 1077 and 1119 (Sigma–Aldrich, Merck Ltd., Bayswater, Australia) density centrifugation. We achieved a purity of 90% for each experiment through morphological staining using May Grünwald Giemsa staining from Sigma–Aldrich Australia (Merck Ltd., Bayswater, Australia). The neutrophils were placed on sterile Ti64 and ZrO_2_ surfaces at 2 × 10^5^ cells per 25 mm^2^ density. Conditioned RPMI (Rosewell Park Memorial Institute) culture media (Thermo Fisher Scientific Ltd., Waltham, MA, USA) was harvested after 1 h, 2 h, and 4 h to measure the amount of neutrophil elastase (NE) and matrix metallopeptidases (MMP-8 and 9) by Enzyme-Linked Immunosorbent Assay-ELISA (R&D invitro Technologies, Australia).

Morphologic analysis of neutrophil by NETosis was conducted following 2 h, 4 h and 8 h of culture on the surface of rough and very smooth Ti and ZrO2 discs at a density of 10^5^ cells per 25 mm^2^. An adequate representation of the central and peripheral zones of the biomaterial surfaces was taken using a Nikon Ti2 widefield microscope (Nikon, NY, USA) at 20× magnification. The quantification of NET zones was performed by measurements of the extracellular stained DNA zones using an Olympus FV3000 confocal laser scanning microscope (Olympus, Tokyo, Japan) and cell profiler software (Broad Institute-MIT, Cambridge, MA, USA), where extracellular DNA was stained blue with 4′,6-diamidino-2-phenylindole (DAPI, ThermoFisher Scientific, Franklin, MA, USA) and neutrophils’ surface was stained pink with CD16b (Thermo Fisher Scientific, Waltham, MA, USA). The software traced the area covered by the extracellular DNA (shown in blue) surrounded by CD16b (shown in pink) and measured the total area per stack in μm^2^. Additionally, the software provided the circularity of the neutrophil DNA on each sample, where a value of 1 represents a perfectly circular object.

Statistical analysis was conducted using IBM SPSS 20 software (IBM, NY, USA), and data were compiled as mean ± SD. Single-factor ANOVA was used to attest the difference between groups at *α* = 0.05. Multiple comparisons were performed using Turkey’s HSD test.

## 3. Results

**Figure 1:**
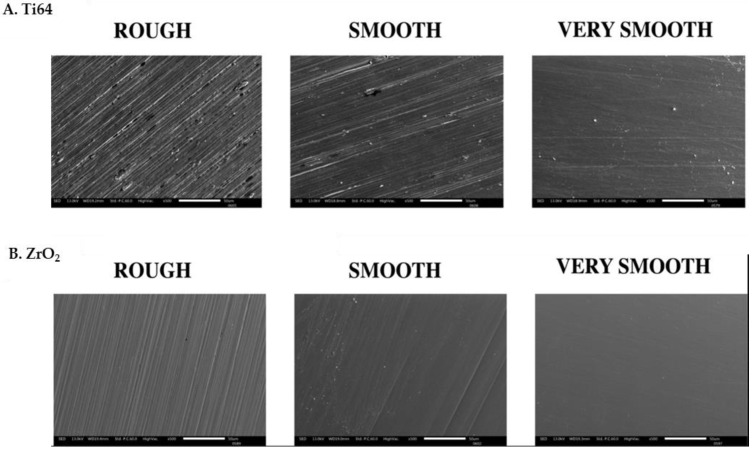
The morphology of the surfaces analysed by scanning electron microscope. (**A**) Ti64; (**B**) ZrO_2_. Scale bar at 50 μm.

The biomaterial surface arithmetical mean height descriptor ‘Ra’ revealed higher values for titanium oxide samples than for ZrO_2_ sources. Ti64-R (3.5 ± 0.06 µm) and ZrO_2_-R (3.2 ± 0.07 µm) surfaces revealed higher values than Ti64-S (1.5 ± 0.04 µm) and ZrO_2_-S (1.1 ± 006 µm) surfaces, which were higher than Ti64-VS (0.05 ± 0.002 µm) and ZrO_2_-VS (0.02 ± 0.005 µm) sources. Rq values were also higher with rougher substrates and generally higher with titanium oxide than the ZrO_2_ substrates. Conversely, Rz values were lower in the titanium oxide substrates. However, rough surfaces demonstrated higher values than smooth and very smooth surfaces. All three roughness descriptors are disclosed in Table 1. Figure 1 represents the surface morphology of the surfaces analysed by scanning electron microscope

**Figure 2:**
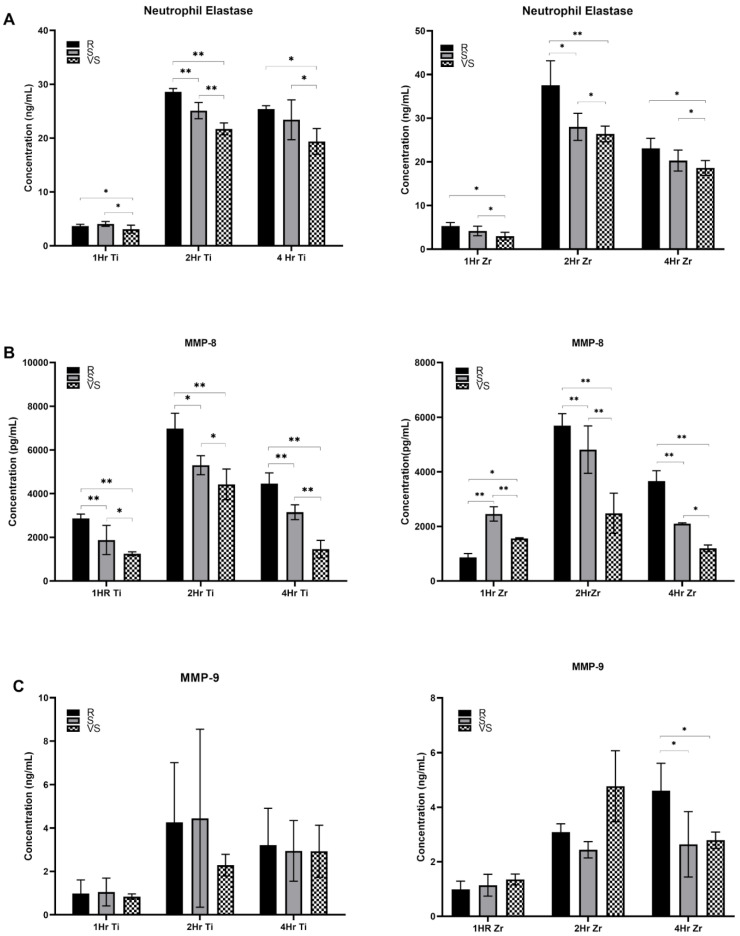
Neutrophil enzyme excretion between Ti64 (Ti) and ZrO_2_ (Zr) surfaces at 1 h, 2 h, and 4 h (**A**) Neutrophil elastase, (**B**) MMP-8, (**C**) MMP-9. * *p* ≤ 0.05; ** *p* ≤ 0.001.

The concentration of neutrophil elastase in the culture supernatant was very low (Figure 2) after 1 h of neutrophil exposure to the Ti64 and ZrO_2_ surfaces for all three roughness levels under analysis. After 2 h, secretion of elastase was higher when neutrophils were exposed to rough titanium oxide and ZrO_2_ surfaces in comparison with smooth (Ti64-S: *p* < 0.001 and ZrO_2_-S: *p* ≤ 0.001) and very smooth surfaces (*p* ≤ 0.001). At 4 h, Ti64 (*p* ≤ 0.007) and ZrO_2_ (*p* = 0.056) rough surfaces again induced higher levels of secretion than very smooth surfaces. However, no significant differences were detected when compared with Ti64 and ZrO_2_ smooth surfaces (*p* = 0.05). ZrO_2_ rough surfaces induced higher levels of elastase secretion after 1 h (*p* = 0.011) and 2 h (*p* = 0.024). However, after 4 h, titanium oxide-based surfaces induced higher levels of elastase (*p* = 0.035).

**Figure 3:**
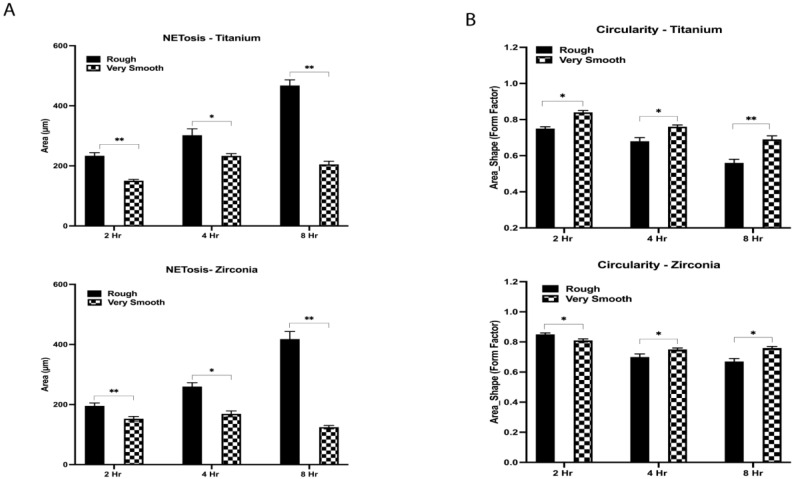
Comparison of (**A**) NET area formed and (**B**) NET circularity (shape) on rough and very smooth titanium (Ti64) VS zirconia (ZrO_2_) surfaces at 2 h, 4 h, and 8 h quantified using cell profiler software. * *p* ≤ 0.05; ** *p* ≤ 0.001.

**Figure 4:**
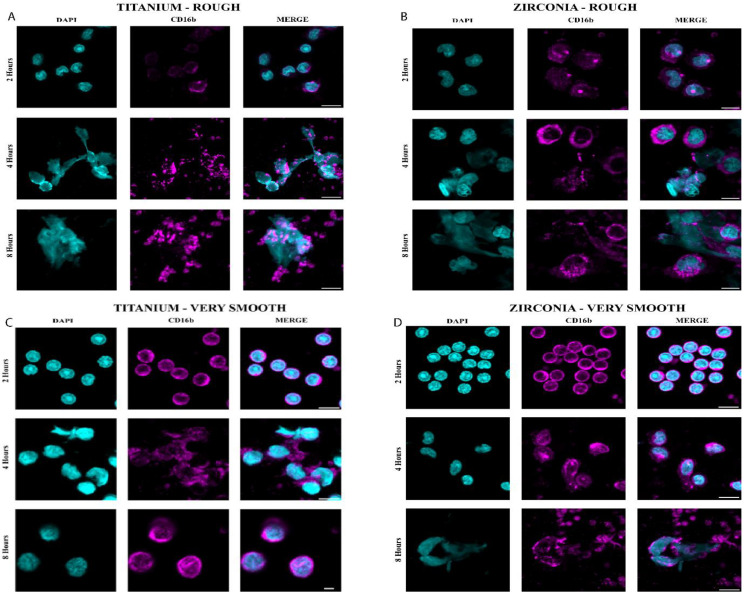
Representative confocal microscopic images of NETs formed on rough (**A**), smooth (**B**), and very smooth titanium (Ti64) (**C**) and zirconia (ZrO_2_) (**D**) surfaces at 2 h, 4 h, and 8 h. The scale bar’s dimension is 20 μm.

More MMP-8 was released by neutrophils exposed for 1 h to Ti64-R surfaces than Ti64-S (*p* ≤ 0.001) and Ti64-VS (*p* ≤ 0.001). The same trend was observed for 2 and 4 h. Neutrophils exposed to the ZrO_2_ surface after 1 h released more MMP-8 on the smooth surface in comparison with rough (*p* ≤ 0.001) and very smooth (*p* = 0.005). After 2 and 4 h, higher levels of MMP-8 were released in ZrO_2_-R surfaces than with S (*p* ≤ 0.001) and VS (*p* ≤ 0.001) surfaces. Generally, Ti64 surfaces induced higher levels of MMP-8 than ZrO2 counterparts after 1 h (*p* ≤ 0.001 ZrO2-R and *p* = 0.017 ZrO2-S), 2 h (*p* = 0.011 ZrO_2_-R, *p* = 0.004 ZrO_2_-S, and *p* = 0.005 ZrO_2_-VS) and 4 h (*p* = 0.004 ZrO_2_-R and *p* ≤ 0.001 ZrO_2_-S). MMP-9 levels were higher following culture on ZrO_2_-R surfaces than on S (*p* = 0.01) and VS (*p* = 0.017) surfaces after 4 h. No significant difference between Ti64 and ZrO_2_ substrates was observed in neutrophil MMP-9 secretion levels (*p* > 0.05). Neutrophils exposed for 2 h to Ti64-R and ZrO_2_-R surfaces revealed swollen and elongated nuclei with larger DAPI-stained areas than observed in Ti64-VS (*p* ≤ 0.001) and ZrO_2_-VS (*p* ≤ 0.001) surfaces. No signs of NETosis were seen on VS surfaces, where neutrophil nuclei were compact and round. Ti64-R (*p* < 0.003) and ZrO_2_-R (*p* < 0.018) surfaces induced larger areas of NETs than VS surfaces after 4 h (Figure 3). Enlarged nuclei oozing out of the neutrophils were observed with R surfaces, whilst elongated neutrophils were observed when in contact with VS surfaces (Figure 3). Significant NET zones and vast web-like structures were seen in Ti64-R (*p* ≤ 0.001) and ZrO_2_-R (*p* ≤ 0.001) surfaces after 8 h (Figure 3 and Figure 4). VS surfaces revealed smaller zones of NETs. Ti64-R (*p* < 0.038) and Ti64-VS (*p* ≤ 0.001) induced larger areas of NETs than ZrO_2_ counterparts. Ti64-VS surfaces presented larger zones of NETs than ZrO_2_-VS (*p* ≤ 0.001).

## 4. Discussion

The mechanobiological modulation of immune cells has been explored in macrophages, the most representative cells from the innate system, due to their significant ability to orchestrate the inflammatory response and healing process. However, neutrophils are the first cells en masse recruited to arrive at implantation sites, and their ability to be physically modulated has not been fully delineated. In this study, we used a range of surface roughness in two common implant biomaterials to analyse its influence on the enzyme secretory ability of neutrophils. We quantified one representative enzyme from the three main granule classes of neutrophils released during degranulation events. Elastase secretion suggests a direct response from neutrophils when faced with rougher surfaces of Ti64 or zirconium dioxide substrates. NE values indicate that higher amounts of the enzyme were released after 2 and 4 h of exposure to the Ti64-R and after 2 h of the ZrO_2_-R surfaces. Hence, neutrophils were less responsive regarding elastase secretion when interacting with smooth and very smooth topographies regardless of the surface substrate. An exemption was observed after 4 h of exposure of neutrophils to the ZrO_2_-S surface, where its elastase secretion was higher than observed on ZrO_2_-R sources. A possible explanation for this finding could be the significant variability of the peaks and valleys pointed out by the Rq and Rz vertical descriptors across the roughness profile of ZrO_2_ surfaces, as seen in Table 1. Similar peaks and valleys are expected along ZrO_2_-R and ZrO_2_-S compared to Ti64 counterparts. Although the Rz descriptor provides an insight regarding the difference between the highest peak and deepest valley from the whole profile, Rz values from ZrO_2_-R and ZrO_2_-S profiles confirm a higher variability and the presence of higher peaks and more profound valleys in ZrO2 surfaces in comparison with Ti64 ones. Although the response of neutrophils against roughness is not well-documented in ZrO_2_-based surfaces, their response against Ti64 substrates has already been presented by different authors [12,13,20,21,22]. Some studies revealed higher recruitment and secretion of elastase and MMP when neutrophils were exposed to smooth surfaces (Sa between 0.5 and 1.5 µm), which is in agreement with our results until the first hour of incubation regarding elastase and MMP-8 and MMP-9 [20]. Over time, neutrophils became more responsive in a time-dependent manner to roughness in terms of elastase and metalloproteinase secretion, and higher levels of elastase and MMP-8 were secreted at 4 h of incubation and MMP-9 after 1 and 2 h of incubation.

Regarding ZrO_2_ substrates, neutrophils exposed to ZrO_2_-R surfaces secreted higher levels of elastase for an hour and at 2 h of incubation, higher amounts of MMP-8 after 2 h and at 4 h and released more MMP-9 at 4 h of incubation. As the response of neutrophils exposed to ZrO_2_ surfaces has never been presented, we established a comparison with Ti64 surfaces at similar roughness levels [20]. Hence, neutrophils showed reactiveness and higher levels of elastase were secreted on ZrO_2_-S surfaces than on ZrO_2_-R surfaces at 4 h. Although this behaviour contrasts with our general findings, other studies have already stated that neutrophil expression was modulated by titanium oxide sources with similar roughness patterns [20]. Regardless, the reason for such discrepancy is still unknown. It could result from the hydrophobic nature of our surfaces, as they were manufactured by milling process instead of acid etching or nitrogen-controlled atmosphere storage protocols. Lower levels of elastase were also identified after one hour on both Ti64 and ZrO_2_ surfaces at the different roughness levels under evaluation. It is coherent with the complexity of the NETosis pathway, which usually demands around 4 h from the decondensation of chromatin until the release of large extracellular web-like structures [15].

Neutrophils usually are activated towards phagocytosis or NET release pathways. Their decision directly influences the resolution of chronic inflammation and the prevention of any unnecessary immune response that could lead to aberrant NETosis [11]. Our results demonstrated an evolution of the neutrophils’ morphology and activation of the NETosis pathway when exposed to different roughness patterns over time. Although at 2 h, no signs of NETosis were expected, elongated and swollen nuclei were observed in Ti64-R and ZrO_2_-R surfaces when compared to Ti64-VS and ZrO_2_-VS substrates, where neutrophils were still shown rounded and compacted morphology. As time increased to 4 h, neutrophils exposed to Ti64-R and ZrO_2_-R surfaces revealed enlarged nuclei oozing out of cells and larger areas usually associated with NET formation, whilst cells in touch with Ti64-VS and ZrO_2_-VS revealed a transition to elongated morphology. At the end of 8 h, neutrophils in contact with Ti64-R and ZrO_2_-R substrates showed mature NET structures extruded as fibrillary networks, while even Ti64-VS and ZrO_2_-VS presented traces of very tiny zones of NET formation.

Herein, rough Ti64 and ZrO_2_ surfaces stimulated higher NET expression. Similar to our findings, Vitkov et al. [19] sandblasted large-grit acid-etched (SLA) triggered histone citrullination and NET release. In contrast to our findings, ref. [20] showed a small area of NET formation on rough Ti64 surfaces compared to smooth ones. The reason for such discrepancy is unknown. We believe that the strong activation of the azurophilic and specific granules is related to a high ROS production and, consequently, might have induced a higher NET formation. However, it is important to note that factors other than surface roughness can affect neutrophil functions, such as surface chemistries and the crystalline structure of the native titanium oxide layer, which could partially affect our results. As our samples were only polished to achieve uniformity after the milling procedures, the native oxide layer from the Ti64 substrates is expected to be in amorphous state with elemental composition following the titanium substrate underneath with a variant ratio of oxygen and aluminium, titanium, and traces of vanadium. Conversely, the ZrO_2_ substrate presents either a tetragonal or cubic crystalline structure depending on the level of translucency demanded during clinical applications. The tetragonal phase is usually stabilised by yttrium oxide with variable contents of aluminium oxide as a dopant. Thus, despite the yttrium oxide content, the elemental composition along the zirconium oxide surface comprises mostly zirconium, oxygen, yttrium, and aluminium. Although the influence of the elemental composition of the Ti64 and ZrO_2_ across the neutrophil activation and secretion capacity is still covered [7], this manuscript presents interesting insights regarding the influence of surface topography and different roughness levels on the neutrophils’ granule release and NETosis. Further analyses are being conducted and will be necessary to better understand the interplay and synergic interaction of the surface chemistry upon the physical properties of either Ti64 and ZrO_2_ substrates along the neutrophils’ activities involved in degranulation and NETosis.

## 5. Conclusions

Our findings suggested that rough Ti64 and ZrO_2_ surfaces triggered an increased secretion of NE, MMP-8, and NET formation from neutrophils compared to smooth surfaces. Also, rough Ti64 surfaces induce a higher neutrophil elastase expression and enhance NET formation more than ZrO_2_ surfaces.

## Figures and Tables

**Table 1:** Surface topography properties (µm).

Group	Ra	Rq	Rz
**TiO_2_**			
Rough	3.5 ± 0.06	4.17 ± 0.16	18.67 ± 0.35
Smooth	1.5 ± 0.04	1.71 ± 0.35	6.87 ± 0.24
Very Smooth	0.05 ± 0.002	0.55 ± 0.03	2.93 ± 0.51
**ZrO_2_**			
Rough	3.2 ± 0.07	4.71 ± 0.18	22.30 ± 0.12
Smooth	1.1 ± 0.06	1.57 ± 0.37	7.19 ± 0.18
Very Smooth	0.02 ± 0.005	0.03 ± 0.08	3.9 ± 0.21

Ra: mean roughness; Rq: root means square roughness; Rz: mean maximum height of the profile.

## Data Availability

The original contributions presented in the study are included in the article, further inquiries can be directed to the corresponding author.
